# Dasatinib Nanoemulsion and Nanocrystal for Enhanced Oral Drug Delivery

**DOI:** 10.3390/pharmaceutics14010197

**Published:** 2022-01-15

**Authors:** Chuanqi Wang, Manting Wang, Peng Chen, Jiexin Wang, Yuan Le

**Affiliations:** 1 State Key Laboratory of Organic-Inorganic Composites, Beijing University of Chemical Technology, Beijing 100029, China; 2017200053@mail.buct.edu.cn (C.W.); 2019410003@mail.buct.edu.cn (M.W.); chentaek@163.com (P.C.); wangjx@mail.buct.edu.cn (J.W.); 2 Research Center of the Ministry of Education for High Gravity Engineering and Technology, Beijing University of Chemical Technology, Beijing 100029, China

**Keywords:** dasatinib, nanoemulsion, nanocrystal, high-gravity technology, in vitro performances

## Abstract

In this work, dasatinib (DAS) nanoemulsion and nanocrystal are produced by high-gravity technology that approaches to practical mass production. The drug nanoformulations were systematically characterized and evaluated. At a low high-gravity level (β) = 47, nanoemulsion droplets were 16.15 ± 0.42 nm with a PDI of 0.122 ± 0.021. The nanoemulsion’s size and active pharmaceutical ingredient (API) content remained stable at long-term (4 months) freeze–thaw and dilution experiments. At a high β = 188, the as-prepared nanocrystal was lamellar with a short diameter of about 200 nm and a long diameter of about 750 nm. In vitro performances demonstrated the nanoemulsion displayed higher cytotoxicity on MDA-MB-231 tumor cells, Caco-2 cell permeability and drug release than that of the nanocrystal, indicating that nanoemulsion should be an ideal alternative for dasatinib oral administration.

## 1. Introduction

Oral administration is the preferred route for drug delivery due to its painless convenience and cost effectiveness [1,2,3]. However, about 40% of the new chemical entity drugs are insoluble in water, which usually leads to low oral bioavailability and poses a huge challenge for new drug development [4]. Consequently, increasing the solubility of drugs in water to improve their oral bioavailability is a sticking point in the development of novel pharmaceutical formulations. The pharmaceutical industry has shown an increasing desire to formulate poorly water-soluble drugs as nanosized formulations with the goal of improving dissolution rate, enhancing bioavailability, eliminating food effects, and improving efficacy and safety. Nanoemulsion and nanocrystal formulations have proved effective to improve the oral bioavailability of hydrophobic drugs with an increasing dissolution rate, eliminating food effects, and improving efficacy and safety [5,6,7,8,9,10]. Drug nanocrystals, such as sirolimus (Rapamune^®^), aprepitant (Emend^®^), fenofibrate (Tricor^®^) and drug nanoemulsions, i.e., cyclosporine (Neoral^®^, Gengraf^®^), saquinavir (Fortovase^®^), ritonavir (Norvir^®^), have been approved by the FDA for clinical application [11,12].

Dasatinib, a class II drug in biopharmaceutical classification with low solubility and high permeability, is a kind of multi-targeted oral tyrosine kinase inhibitor for the treatment of chronic myelogenous leukemia and Philadelphia chromosome-positive lymphoid leukemia [13]. Currently, there are two commercial DAS tablets that have been approved for clinic use. One is SPRYCEL^®^ produced by Bristol–Myers Squibb (BMS) with the crystal form of MH H1-7, approved in 2006 by the FDA. The other is YINISHU^®^ produced by CHIA TAI TIANQING with the crystal form of AH N-6 [14], approved in 2013 by the CFDA. AH N-6 displays higher solubility and lower stability than that of MH H1-7 [15]. The solubility of DAS is closely related to the pH of solution. The data provided by BMS show that DAS displays higher solubility at pH lower than 4, and its solubility is 205 μg/mL at pH 4.28, while less than 1 μg/mL at pH 6.99 [16]. The bioavailability of DAS is only 14–34% in mammals [17] due to incomplete absorption and high first-pass effect caused by low solubility in small intestine [18,19,20].

Nanonization strategies have been applied to improve the drug release as well as oral bioavailability of DAS. Reddy [21] employed the wet granulation method to prepare DAS tablets with enhanced immediate release using croscarmellose. Maheswari et al. [22] prepared spherical micelles of DAS encapsulated by sodium lauryl sulfonate and lactose monohydrate applying spray-drying in order to increase its solubility at pH > 4. However, the amount of the excipients is far beyond the safe dosage given by the FDA and the average size of the product is larger than 2.5 μm with severe agglomeration. Begum et al. [23] prepared DAS lipid nanoparticles using high-pressure homogenization. The release of DAS could be enhanced by adding lecithin and poloxamer into the formulation of lipid granules. The as-prepared lipid suspensions ranged from 94 to 800 nm. However, after drying, the particles agglomerated to hundred microns, which greatly weakened the solubilization of nano lipid DAS.

Therefore, it is necessary to develop novel nanoformualtions to improve the oral bioavailability of DAS. Nanoemulsions and nanocrystals are considered as ideal alternatives for the oral administration of the drug because they exhibit various advantages, such as a high solubilization capacity for both hydrophilic and hydrophobic drugs [24,25] and the ability to improve lymphatic absorption, hence avoiding first-pass metabolism and enhancing bioavailability [26,27].

In addition, approaches to prepare nanoformulations have attracted much more attention. Up to now, commercial formulations of nanocrystals have been produced by breaking down large particles using a relatively simple and efficient top-down process, such as wet ball milling and high-pressure homogenization [28,29]. Commercial formulations of nanoemulsions have been made by using low energy methods involving spontaneous emulsification and phase inversion [30].

It is a strong innovation to develop general efficient techniques for the production of pharmaceutical nanoformulations. High-gravity technology, implemented by a rotating packed bed (RPB), has been used as an effective process intensification technology, which can generate an acceleration of 1–3 orders of magnitude greater than the gravitational acceleration of the Earth. The fluids going through the packing of RPB are spread or split into very fine droplets, threads, and thin films by strong shear force, resulting in a significant intensification of micro-mixing and mass transfer between the fluid elements and, hence, benefiting from the form of uniform concentration distribution. RPB, with an ultrashort residence time (<1 s) of reactants, has proved to be an ideal device for the preparation of nanoparticles and successfully applied in the pharmaceutical industry [31,32,33,34].

The main objective of this work is to produce a DAS nanoemulsion and nanocrystal using a high-gravity technique to enhance its oral bioavailability. Formulation and process parameters are investigated and optimized. In vitro drug release, Caco-2 cell permeability and cytotoxicity on MDA-MB-231 tumor cells of both nanoformulations are evaluated and compared.

## 2. Materials and Methods

### 2.1. Materials

DAS, glyceryl triacetate, isopropyl myristate, oleic acid, and Triton X-100 were purchased from Shanghai Macklin Biochemical Co., Ltd. (Shanghai, China). RH 40 and SL-15 were purchased from BASF (Ludwigshafen, Germany). Ethyl oleate, Tween-20, Tween-80, and mannitol were obtained from Aladdin Bio-Chem Technology Co., Ltd. (Shanghai, China). Ethyl acetate, ethanol, PEG 200, PEG 400, and N,N-dimethylformamide (DMF) were purchased from Beijing Chemical Factory (Beijing, China). 1,2-propanediol was purchased from Shanghai Yuanye Bio-Technology Co., Ltd. (Shanghai, China). Purified water was bought from Hangzhou Wahaha Group Co., Ltd. (Hangzhou, China). 4,6-diamidino-2-phenylindole (DAPI), dimethyl sulfoxide (DMSO), and 3-(4,5-dimethyl-2-thiazolyl)-2,5-diphenyl tetrazolium bromide (MTT) were purchased from Sigma-Aldrich (Shanghai, China). Dulbecco’s modified eagle medium (DMEM), and phosphate-buffered solution (PBS) were obtained from Gibco (Carlsbad, CA, USA). Caco-2 cells were provided by the Peking University Health Science Center. MDA-MB-231 cells were provided by The Chinese Academy of Sciences cell bank.

### 2.2. Methods

#### 2.2.1. Equilibrium Solubility Studies

Oil-phase components for the development of the DAS nanoemulsion were selected based on equilibrium solubility studies. Excess DAS was added to oil phase and kept on a water bath shaker at 25 °C for 24 h after vortex mixing for 10 min. As-obtained samples were centrifuged at 12,000 rpm for 10 min. The supernatant was analyzed using a validated high-pressure liquid chromatography (HPLC) method.

#### 2.2.2. Preparation of the DAS Nanoemulsion

**Figure 1:**
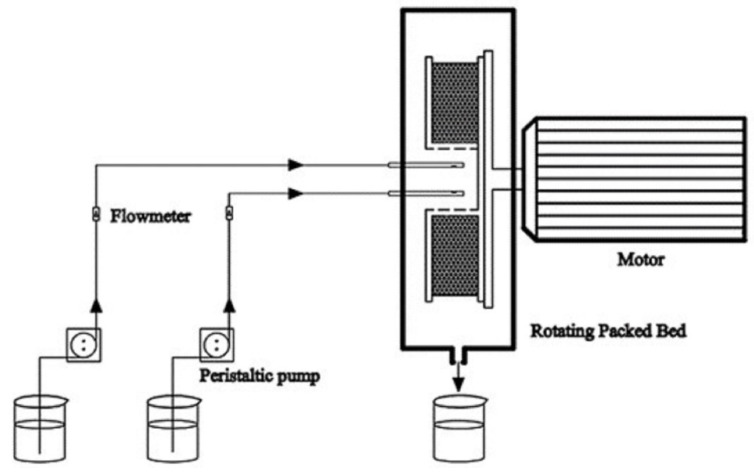
Schematic diagram of the preparation procedure.

On the basis of equilibrium solubility studies (pseudo-ternary phase diagram) and the measurement of the DAS solubility, the blank nanoemulsion was constructed by mixing oleic acid/RH-40/1,2-propanediol/purified water = 2.25:15.17:7.58:75 (*w*/*w*/*w*/*w*). The oil phase, surfactant and co-surfactant were mixed to form the mixed oil phase. A saturated DAS oil solution was first configured and then the excess DAS solids were removed by centrifugation at 5000 rpm for 10 min. The preparation procedure of the DAS nanoemulsion was shown in Figure 1. In details, the mixed oil phase and purified water were, respectively injected into an RPB by peristaltic pumps at the feeding rates of 200 mL/min and 600 mL/min at the temperature of 20 °C. The DAS nanoemulsion was collected from the outlet continuously. For contrast, a conventional stirring tank reactor (STR) was chosen to prepare another group of DAS nanoemulsion. Mixed oil phase was quickly added to purified water at 20 °C and stirred at 500 rpm for 15 min.

#### 2.2.3. Preparation of the DAS Nanocrystal

DAS was added to DMF to obtain the solvent phase (50 mg/mL). Mannitol was used as excipient and added to purified water to obtain the antisolvent phase (2.5 mg/mL). The two phases were, respectively injected into an RPB reactor at the feeding rates of 30 mL/min and 600 mL/min at a temperature of 25 °C. The DAS recrystallization dispersion was collected continuously at the outlet, and solid nanocrystals were obtained by lyophilization.

### 2.3. Characterization

#### 2.3.1. Droplet Size and Polydispersity Index

The dynamic light scattering (DLS) technique was performed to analyze the droplet size and polydispersity index (PDI) value of DAS nanoemulsion by using a laser particle sizer (Nano-ZS90, MALVERN, Malvern, UK) at 25 °C. Average droplet size was determined by intensity distribution.

#### 2.3.2. TEM and SEM

The morphology of DAS nanoemulsion was examined by a transmission electron microscope (TEM) (H-9500, HITACHI, Tokyo, Japan) with an accelerating voltage of 100 KV. The appropriate amount of nanoemulsion was dripped on fresh copper grids and then observed after drying at room temperature. The morphology of the DAS nanocrystal was examined by scanning electron microscopy (SEM) (JSM-7800F, JEOL, Tokyo, Japan) operated at 5 kV.

#### 2.3.3. In Vitro Drug Dissolution Study

The in vitro drug release of DAS samples was determined in a dissolution tester (Vision G2 Elite 8, HANSON, Los Angeles, CA, USA). A PBS solution (pH 6.8) with 1% Triton X-100 was used as release medium. The samples loaded with 20 mg of DAS were placed in treated dialysis bags, and 20 mL release medium was added into each dialysis bag. The dialysis bags were suspended in 900 mL release medium, respectively, stirred at 60 rpm with the temperature of 37 °C. Sampling was at specified time intervals, and meanwhile, an equal amount of fresh release medium was supplemented. The content of DAS in the release medium was determined by HPLC analysis.

#### 2.3.4. In Vitro Cytotoxicity Assay

The cytotoxicity of DAS nanoformulations with different concentrations (0.1, 0.5, 1, 3, 5, 10, 15, 30 μg/mL) against MDA-MB-231 cells were determined by the MTT method. MDA-MB-231 cells were incubated in DMEM medium containing 10% FBS and 1% penicillin/streptomycin (complete medium) and seeded in 96-well microplates at a density of 4 × 10^3^ cells/well and incubated for 12 h. DAS nanoformulations diluted by PBS (pH = 6.8) were then added into wells and equal volume of PBS was used as the control. After 12 h and 24 h incubation, respectively, 20 μL MTT solution was added. After being incubated for 4 h, the medium was removed and 100 μL DMSO was added into each well to dissolve the formazan produced by the active cells. The absorbance at 570 nm was measured by a microplate reader and the cell viability was calculated by Equation (1):

(1) $$\mathrm{Cell}\;\mathrm{viability}=\frac{{\mathrm{OD}}_{\mathrm{sample}\;}{-\;\mathrm{OD}}_{\mathrm{blank}}}{{\mathrm{OD}}_{\mathrm{control}}{\;-\;\mathrm{OD}}_{\mathrm{blank}}}$$

where OD_sample_, OD_control_, OD_blank_ are the optical density of experimental group, control group and non-cells group, respectively.

#### 2.3.5. Cellular Uptake Study

The Neil red-loaded DAS nanoemulsion was added to Caco-2 cells at regular intervals and cultured at 37 °C in an atmosphere of 5% carbon dioxide (CO_2_). The cells were fixed with paraformaldehyde, and the nuclei were stained with DAPI. Confocal laser scanning microscopy (TCS SP5 II, LEICA, Wetzlar, Germany) was performed to observe the cellular uptake.

#### 2.3.6. Transport Study

Before conducting the Caco-2 cell permeability test, the trans-epithelial electrical resistance (TEER) value was measured, and the appropriate drug concentration was determined to ensure the integrity of Caco-2 cell monolayers. The nanoemulsion diluent with drug concentration less than 8 μg/mL had no cytotoxicity (cell viability > 90%) after 4 h for administration. Therefore, to ensure the accuracy of the measurement, all samples in vitro permeability study were tested with a drug concentration of 8 μg/mL. For the transport study from the apical (AP) side to the basolateral (BL) side, the initial 0.5 mL medium at the AP side was replaced with 0.5 mL as-prepared drug-loaded dilution, while 1.5 mL complete medium was added to the BL side, and then incubated at 37 °C in an atmosphere of 5% CO_2_. A total of 0.5 mL samples were taken from the BL side at regular intervals (30, 60, 90, 120, 150, 180, 210, and 240 min), and meanwhile, 0.5 mL fresh complete medium was supplemented. For the transport study from the BL side to the AP side, 0.5 mL drug-loaded dilution was added to the AP side and washed with PBS after incubation for 30 min, and after that, a 0.5 mL fresh complete medium was supplemented, and the initial 1.5 mL medium in the BL side was replaced with 1.5 mL drug-loaded dilution. A total of 0.2 mL samples were taken from the AP side at regular intervals (30, 60, 90, 120, 150, 180, 210, and 240 min) and 0.2 mL fresh complete medium was supplemented, meanwhile. Three parallel experiments were set up, and the concentration of samples was determined by HPLC. The apparent permeability coefficient (P_app_) is calculated by Equation (2), and the efflux rate (ER) is calculated by Equation (3):

(2) $$P_{\mathrm{app}}=\frac{\mathrm{dQ}}{\mathrm{dt}}\cdot \frac{1}{{\mathrm{AC}}_{0}}$$

where P_app_ is the apparent permeability coefficient (cm/s), dQ/dt is steady-state permeation flux (μg/s), A is the surface area of cell monolayer membrane (cm^2^), and C_0_ is the initial DAS concentration on the administration side (μg/mL).

(3) $$\mathrm{ER}=\frac{P_{\mathrm{app}\;\mathrm{BL}-\mathrm{AP}}}{P_{\mathrm{app}\;\mathrm{AP}-\mathrm{BL}}}$$

where P_app BL-AP_ is the apparent transmission coefficient of BL to AP side transport, and P_app AP-BL_ is the apparent transmission coefficient of AP to BL side transport.

## 3. Results and Discussion

### 3.1. Construction of the DAS Nanoemulsion

**Figure 2:**
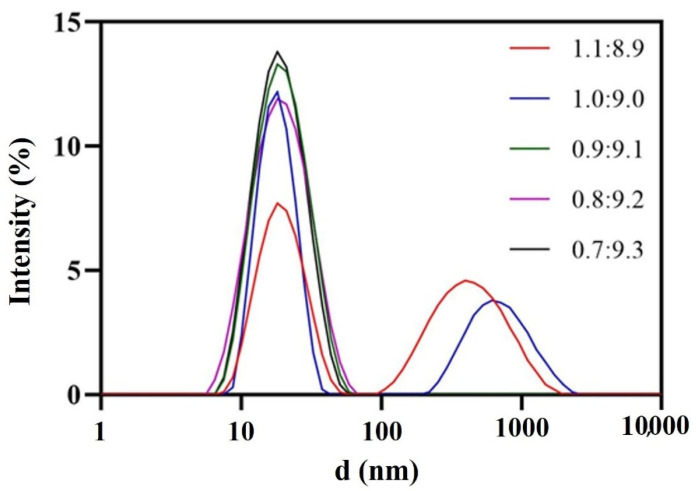
Droplet size distribution at different oil-mixed surfactant ratios.

Oleic acid served as the oil phase due to its higher solvency for DAS (25 °C, 26.66 ± 4.20 mg/mL). The surfactant had a considerable influence on the nanoemulsion. According to Taylor’s equation, which emphasizes the pivotal role of amphiphilic surfactants in reducing interfacial tension and enhancing emulsion stability, RH 40 was chosen after being compared with other common surfactants with the aid of the pseudo-ternary phase diagram and FDA’s excipient dosage data. 1,2-propanediol was selected as co-surfactant, the ratio of surfactant to co-surfactant (Km) was 2:1 and the amount of surfactant and co-surfactant were minimized as much as possible. Consequently, 0.9:9.1 was chosen as the ratio of oil phase to mixed surfactant (Figure 2). Due to the comprehensive consideration of the droplet size and drug-loading capacity of the nanoemulsion, the ratio of mixed oil phase to aqueous phase was 1:3. Finally, the blank nanoemulsion was constructed by oleic acid/RH-40/1,2-propanediol/purified water = 2.25:15.17:7.58:75 (*w*/*w*/*w*/*w*). Drug loading could reach 3.5 mg/mL, which was more than 1700 folds of the solubility of MH H1-7 DAS in water and 500 folds of the solubility of AH N-6 DAS in water.

**Figure 3:**
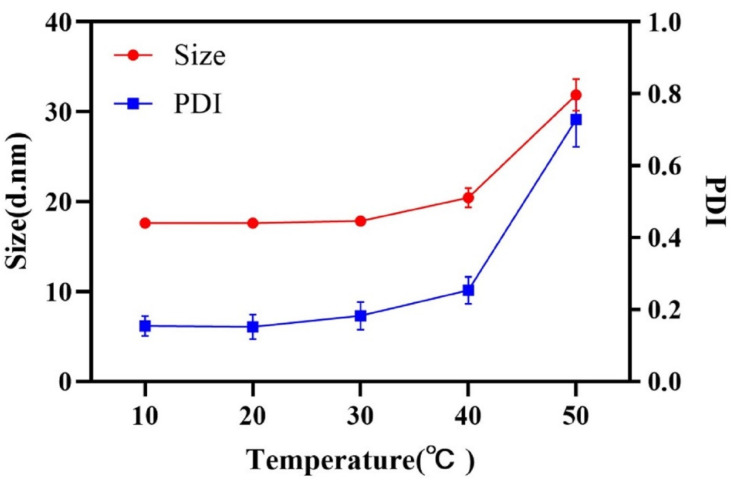
Droplet size and PDI at different emulsification temperatures.

The hydrophilicity of surfactants, changing with the temperature, has a significant influence on emulsification process. The effect of temperature on the droplet size was investigated and shown in Figure 3. A lower temperature was beneficial to obtain a nanoemulsion with small droplet size and narrow size distribution. The hydrophilicity of RH 40 strengthened with the decrease in temperature, resulting in the remarkable reduction in oil–water interfacial tension, so as to be able to obtain a stable nanoemulsion with a small droplet size. A temperature of 20 °C, which exhibited a strong connection at the oil–water interface utilizing RH 40, was selected to prepare the nanoemulsion.

Fixing the emulsion temperature at 20 °C, RPB was introduced to intensify the emulsification process. High-gravity level (β) represents the intensification degree of mass transfer and micro-mixing [17] had some influence on the droplet size. β is determined by Equation (4):

(4) $$\beta =\frac{{\left(2\pi n\right)}^{2}}{g}\frac{\int_{r_{1}}^{r_{2}}{2\pi r}^{2}\mathrm{dr}}{\int_{r_{1}}^{r_{2}}2\pi \mathrm{rdr}}=\frac{{8\pi }^{2}n^{2}{(r}_{1}^{2}{+r}_{1}r_{2}{+r}_{2}^{2})}{{3(r}_{1}{+r}_{2})g}$$

Here, n is the rotor speed of RPB (rpm), g is the earth gravitational acceleration (9.8 m/s^2^), r_1_ is the inner radius of packing (m), and r_2_ is external radius of packing (m).

**Figure 4:**
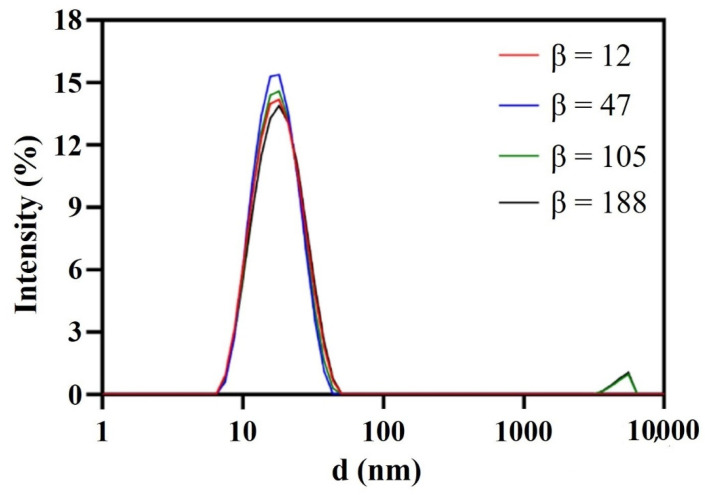
Droplet size distribution of nanoemulsions prepared by RPB with different β.

**Figure 5:**
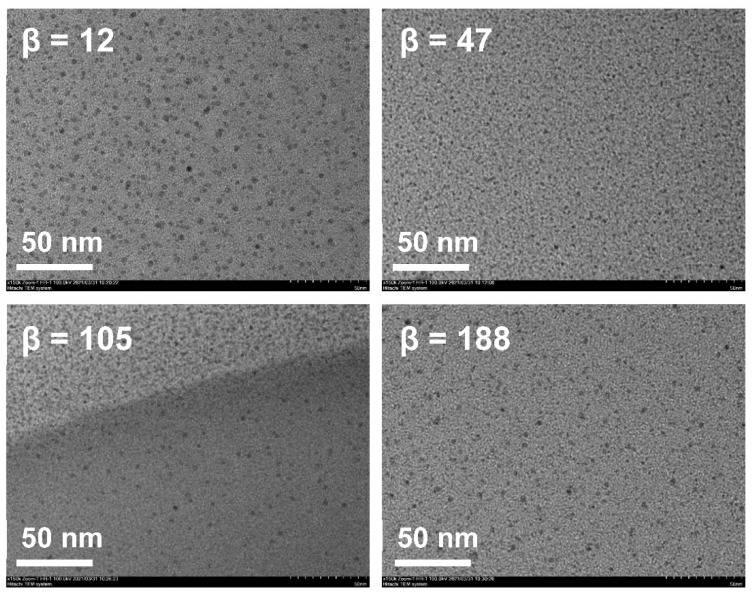
TEM of nanoemulsions prepared by RPB with different β.

As shown in Figure 4 and Figure 5, β hardly impacted the droplet size; when increased from 12 to 47, the size decreased a little and the droplet distribution became more concentrated; with the further increase in β, the size increased slightly and the size distribution remained narrow. In RPB, the oil droplets were too soft to resist the shear force generated by the high-gravity field, so they were easily broken up into tiny droplets. Nevertheless, as fluid turbulence became more intense due to a further β increase, the surface of tiny droplets was damaged and failed to recover in time, causing droplet adhesion and formation of slightly larger droplets. Generally, β showed no significant effect on droplet size due to the homogeneous distribution environment provided by RPB reactor. The resultant nanoemulsion at the lower β of 47 exhibited the most narrow size distribution, with sizes of 16.15 ± 0.42 nm and PDI of 0.122 ± 0.021.

**Figure 6:**
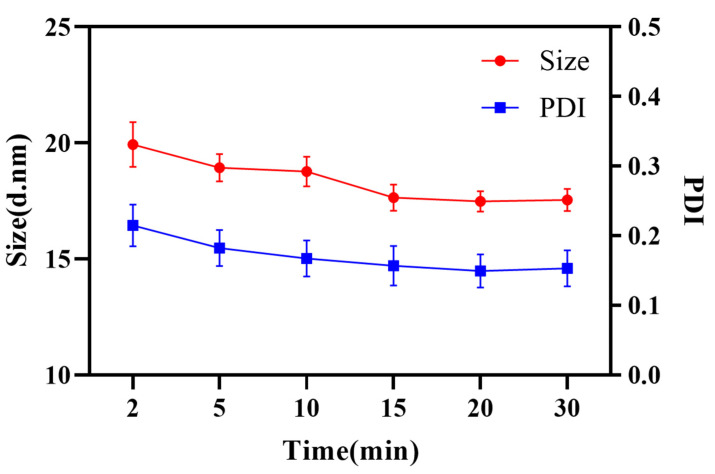
Droplet size and PDI at different emulsification times.

Meanwhile, the STR emulsion process was investigated. The emulsification time imposed on both droplet size and PDI. Evidently, enough time was necessary to complete micro-mixing of oil and water. As shown in Figure 6, it took 15 min to fulfill the micro-mixing and obtain a stable nanoemulsion with an average size of 17.69 ± 0.62 nm and PDI of 0.171 ± 0.048. In comparison, the micro-mixing of oil and water could finish immediately in RPB, which improved the efficiency and reduced the energy consumption as well as realizing the continuous production of the nanoemulsion.

### 3.2. Stability Study of the Nanoemulsion

**Figure 7:**
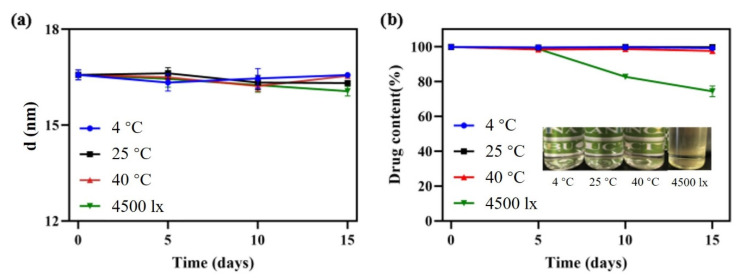
Droplet size change (**a**) and appearance and content change (**b**) under different storage conditions.

Stability is not only an important index to evaluate the quality of the nanoemulsion, but also a guidance for storage, transportation and utilization in practical applications. Firstly, the effects of various influencing factors on stability for 2 weeks were tested. Temperature had little effect on droplet size and drug content as shown in Figure 7. The phenomenon was attributed to the intense micro-mixing and uniform concentration field generated by RPB, greatly reducing the interfacial tension between oil and water. Therefore, the droplet accumulation caused by the Ostwald ripening (OR) [35] and particle migration and coalescence (PMC) was greatly decreased, even at low or high temperatures representing ambient conditions. After storage for 2 weeks under a strong light of 4500 ± 500 lx, although the droplet size varied only in a small range, the content of DAS decreased to 74.35% and the appearance changed from colorless to light yellow. The main reason is that oleic acid can be oxidized easily under strong light, which causes the change of DAS content.

Additionally, 4-month long-term stability experiments at 25 °C and 40 °C, representing normal room temperature and high temperature, were evaluated. The sample stored at 25 °C exhibited excellent stability. Its droplet size changed only in a small range, the drug content remained above 98% and appearance remained colorless and transparent. At 40 °C, the drug content decreased by 12.37% and the appearance turned slightly yellow, as a result of the oxidation of oleic acid after long-term storage at a high temperature.

The freeze–thaw stability was tested because the nanoemulsion may undergo rapid changes in ambient temperature during storage and transportation. An as-prepared sample was cyclically exposed to a 4 °C and 40 °C environment. After a 3-cycle freeze–thaw test, the droplet size and DAS content had no significant change (Table 1), indicating that the nanoemulsion could adapt well to ambient temperature.

**Figure 8:**
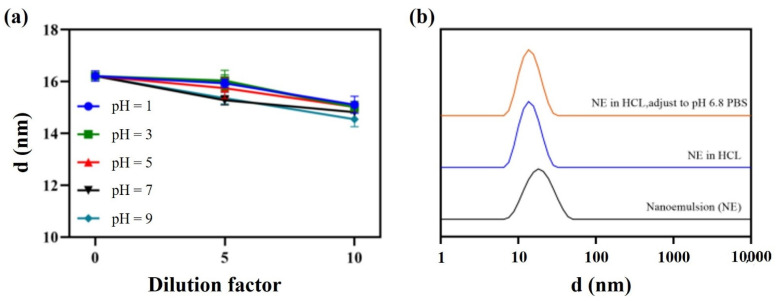
Direct dilution stability (**a**) and pH dynamic dilution stability (**b**).

Furthermore, direct dilution and pH dynamic dilution stability tests were carried out to evaluate the performance of the nanoemulsion in the gastrointestinal tract. The droplet size remained almost the same whether samples were diluted 10 times at a pH range of 1–9 (Figure 8a), or pH changed dynamically from 1.2 to 6.8 (Figure 8b).

### 3.3. Construction of the DAS Nanocrystal

The DAS nanocrystal was prepared by the high gravity antisolvent recrystallization method [32]. DMF was screened as solvent, and water as antisolvent. The ratio of solvent to antisolvent was 1:20 (*v*/*v*), and the concentration of DAS in the solvent phase was 50 mg/mL. Mannitol, a common medicinal lyophilized protective agent with outstanding hydrotropy, was served as excipient. The dosage of mannitol used in antisolvent phase was equal to that of DAS.

**Figure 9:**
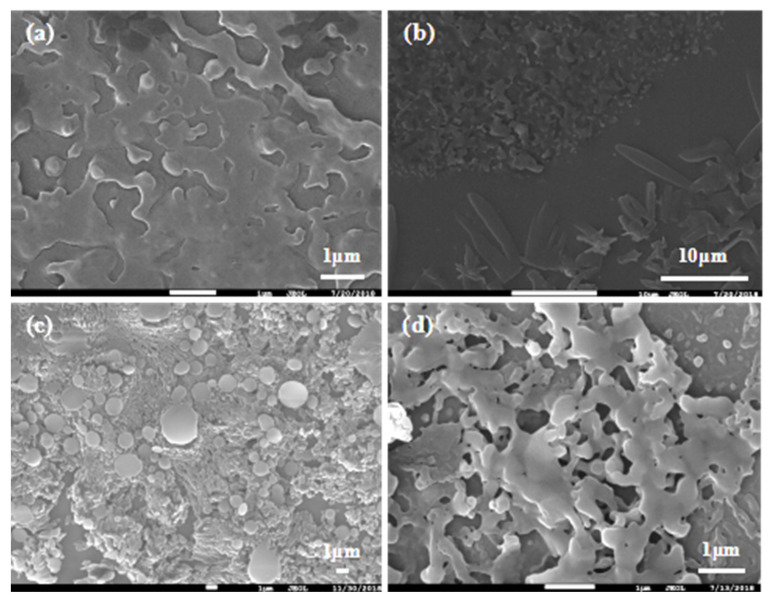
Recrystallization at different β: (**a**) β = 47; (**b**) β = 105; (**c**) β = 188; and (**d**) β = 293.

Similarly, effects of β on particle size were investigated. As shown in Figure 9, β had a small effect on the dispersion of particles. Firstly, particles dispersed gradually with β increase. When fluid turbulence was intense at high β, micro-mixing in RPB was more uniform and the efficiency of interphase mass transfer was higher, contributing to the extremely even dispersion of the crystal nucleus in the droplet microelements. At β = 188, the dispersion of particles was the best and the morphology was relatively regular. Additionally, the particles agglomerated together again when β continuously increased. Shear force became stronger with the further increase in β, leading to a fiercer collision between droplets and the instability of the crystal nucleus. Thereby, particles were inclined to agglomerate and merge.

**Figure 10:**
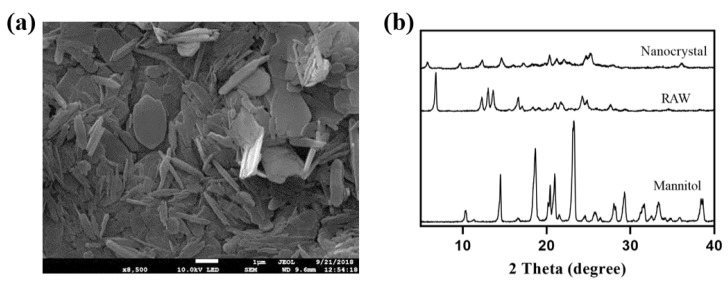
SEM image (**a**) and XRD results (**b**) of nanocrystals obtained by lyophilization.

The nanocrystals obtained by the freeze-drying method were mainly long flakes with a length of 750 nm (Figure 10a), with AH N-6 crystal form (Figure 10b). The intensity reduction and broadening of characteristic peaks at 12.3° and 16.7° were consistent with the theory that particle size and crystallinity decrease after recrystallization.

### 3.4. In Vitro Performanance

**Figure 11:**
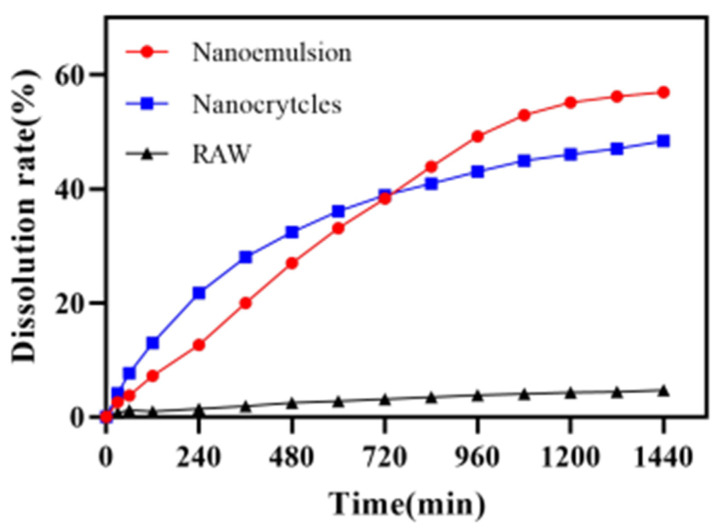
Release curves of the DAS samples.

Figure 11 shows the release profiles of the DAS nanoemulsion, DAS nanocrystal and raw DAS. The results obtained from the dissolution experiments revealed that the dissolution behavior of DAS was significantly enhanced though the preparation of the nanoformulation. The dissolution of the DAS nanoemulsion and DAS nanocrystal reached 56.96% and 48.38%, respectively after cumulative release for 24 h, while that of raw DAS was only 4.72%. The enhancement in dissolution rate of the DAS nanoformulation could be attributed to increased solubility caused by particle size reduction, according to the Ostwald–Freundlich equation [33]. The DAS nanoemulsion had a much better improved solubility than that of the DAS nanocrystal because of its smaller droplets. While the DAS nanocrystal had evident advantages in promoting drug release at the initial stage, because of being different to the DAS nanoemulsion, drug diffusion did not undergo the process from oil phase to external media.

**Figure 12:**
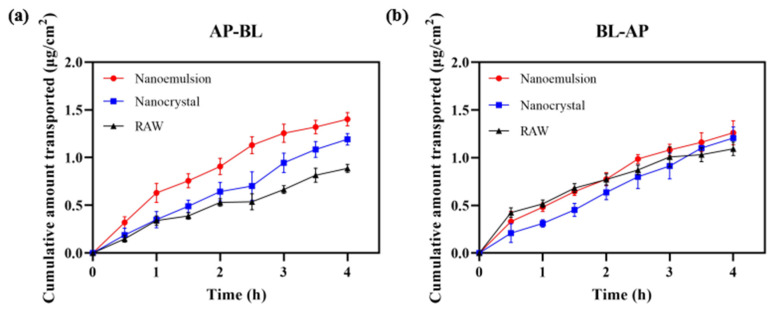
Cumulative transport capacity of different DAS samples in the direction of AP–BL (**a**) and BL–AP (**b**).

Figure 12 displays the permeability of DAS across Caco-2 cell monolayers. When absorptive transport was in the AP–BL (apical to basolateral) direction, the cumulative permeation of DAS was ordered as follows: nanoemulsion > nanocrystal > raw. The nanoemulsion and nanocrystal could smoothly pass through with P_app_ of 1.17 × 10^−5^ cm/s and 0.97 × 10^−5^ cm/s, which were 1.63 times and 1.35 times higher, respectively, than that of the raw DAS (0.72 × 10^−5^ cm/s). In the BL–AP (basolateral to apical) direction, the P_app_ of nanoemulsion, nanocrystal and raw DAS had no significant difference, which were 1.03 × 10^−5^ cm/s, 1.00 × 10^−5^ cm/s and 0.92 × 10^−5^ cm/s (*p* < 0.01), respectively. Although DAS is a kind of BCS II drug with good permeability, it is difficult to pass through small intestinal epithelial cells due to its poor water solubility, resulting in insufficient absorption. The nanoformulation, with a smaller size and higher dissolution, makes it possible to improve cell permeability and finally realize a superior bioavailability. Compared with the nanocrystal, the nanoemulsion formulation had an enhanced permeability on Caco-2 cell monolayers due to the usage of surfactants and the formation of tiny oil-in-water droplets [32,33,34].

**Figure 13:**
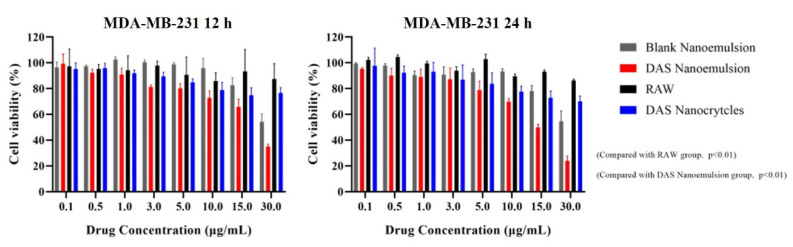
Cell inhabitation rates of the different samples against tumor cells.

The in vitro antiproliferative activities of the DAS nanoformulation and raw DAS on MDA-MB-231 triple negative breast cancer cells were measured using the MTT method (Figure 13). The results showed that the DAS nanoformulation and raw DAS inhibited MDA-MB-231 cell replication in a concentration- and time-dependent manner. Compared to the raw-DAS-treated cells under the same experimental conditions, a significant increase in inhibition rate was observed in the DAS-nanoformulation-treated cells. When incubated for 24 h, the inhibition rate of the cell activity reached 30.08% in the DAS nanoemulsion and 22.33% in the DAS nanocrystal, while it was only 11.51% in the raw DAS. The overall anticancer ability of the raw DAS was very low even increasing drug concentration because its poor solubility delayed the inhibition effect on tumor cells. Blank nanoemulsion almost had no cytotoxicity on MDA-MB-231, indicating that the formulation was safe. In the manner of dissolution and permeability tests, the DAS nanoemulsion exhibited a much better antiproliferative activity than the DAS nanocrystal, which demonstrates that smaller lipid droplets are helpful for cellular uptake.

## 4. Conclusions

In this work, a DAS nanoemulsion and nanocrystal for oral drug delivery were successfully prepared by high-gravity technology, which approaches to practical mass production. DAS nanoemulsion with droplets of about 17 nm remained physicochemically stable under different conditions. The DAS nanocrystal with a particle size of about 750 nm reached a solubility of 28.20 μg/mL in PBS (pH = 6.8) at 37 °C, more than 4-fold than that of the raw DAS. In vitro dissolution, cell permeability and cytotoxicity tests showed that the nanoemulsion and nanocrystal could greatly improve drug release, Caco-2 cell permeability and inhibition on MDA-MB-231 tumor cells. By contrast, the DAS nanoemulsion displayed better in vitro performance than the DAS nanocrystal. Taken together, these results suggest that high-gravity technology can open a new avenue for the continuous manufacturing of promising candidates, DAS nanoemulsion and DAS nanocrystal, for the treatment of chronic myelogenous leukemia and Philadelphia chromosome-positive lymphoid leukemia.

## Figures and Tables

**Table 1:** Freeze–thaw experiment results of the DAS nanoemulsion.

Cycle	Size (nm)	Drug Content (Relative to the Initial %)
Before freeze–thaw test	16.32	100%
1	16.07	100%
2	16.16	100%
3	16.83	100%

## Data Availability

Not applicable.
